# New Appliances and Things Medical

**Published:** 1904-06-18

**Authors:** 


### NEW APPLIANCES AND THINGS MEDICAL.

[We shall be glad to receive at our Office, 28 & 29 Southampton Street, Strand, London, W.C., from the manufacturers, specimens of all new preparations and appliances which may be brought out from time to time.]

### CAMWAL WATERS.

Camwal, Limited, 112 Pembroke Street, Caledonian Road, London, N.

We have received the following samples of these waters, viz., Soda Water, Lithia Water, Seltzer Water, Potash Water, Piperazine Water, and Lemonade in syphons; also Ginger Beer, Lemonade, Ginger Ale, and Soda Water in small bottles. We have examined these waters carefully, and have arrived at the conclusion that they are good reliable waters, and contain the substance stated upon the label. They are well aerated, and speaking generally have a pleasant taste. The piperazine water we regard as likely to be useful in cases of the uric acid diathesis. The solvent action of this substance has been worked out, and compared with that of other uric acid solvents by Tunnicliffe and Rosenheim and others. The solubility of lithium urate as compared wtth sodium and potassium urate is already well known. The objection that by the drinking of these waters only a very small quantity of the solvent is introduced into the system does not seem to us a sound one, in that at present we are quite ignorant of the quantitative pathological relationships of the uric acid salts, and until this is determined the introduction into the system of a uric acid solvent in however small quantity may be of service. In our opinion the samples sent are to be recommended.

### COUGH LINCTUS.

(Evans, Gadd and Co., Limited, 97–100 Fore Street, Exeter.)

We have received two varieties of cough linctus from this firm of druggists, viz., Glycerol Heroin Hydrochloride Co. and Linctus Codeinæ. The active principle of the first is heroin, and it is so arranged that each fluid drachm contains one-sixteenth of a grain of this substance, its action being fortified by senega, wild cherry bark, containing, of course, prussic acid, and balsam of tolu. The dose of this linctus is from half to one fluid drachm. The second preparation, viz., linctus codeinæ co. contains one-eighth of a grain of phosphate of codeine to the fluid drachm, and the dose is given as one-half to one fluid drachm. We have tried both these preparations at one of the London Chest Hospitals, and found them agreeable in taste and satisfactory in action.

